# Evaluation of the long-lasting insecticidal net *Interceptor LN*: laboratory and experimental hut studies against anopheline and culicine mosquitoes in northeastern Tanzania

**DOI:** 10.1186/1756-3305-6-296

**Published:** 2013-10-12

**Authors:** Robert Malima, Patrick K Tungu, Victor Mwingira, Caroline Maxwell, Stephen M Magesa, Harparkash Kaur, Matthew J Kirby, Mark Rowland

**Affiliations:** 1 Amani Medical Research Centre, National Institute for Medical Research, P.O. Box 81, Muheza, Tanzania; 2 Faculty of Infectious and Tropical Diseases, London School of Hygiene & Tropical Medicine, WC1E 7HT London, UK

**Keywords:** Long-lasting insecticidal net, LN, Interceptor LN, *Anopheles gambiae*, *Anopheles funestus*, *Culex quinquefasciatus*, Experimental hut

## Abstract

**Background:**

Long lasting insecticidal nets (LN) are a primary method of malaria prevention. Before new types of LN are approved they need to meet quality and efficacy standards set by the WHO Pesticide Evaluation Scheme. The process of evaluation has three phases. In Phase I the candidate LN must meet threshold bioassay criteria after 20 standardized washes. In Phase II washed and unwashed LNs are evaluated in experimental huts against wild, free flying anopheline mosquitoes. In Phase III the LN are distributed to households in malaria endemic areas, sampled over three years of use and tested for continuing insecticidal efficacy. Interceptor*®* LN (BASF Corporation, Germany) is made of polyester netting coated with a wash resistant formulation of alpha-cypermethrin.

**Methods:**

Interceptor LN was subjected to bioassay evaluation and then to experimental hut trial against pyrethroid-susceptible *Anopheles gambiae* and *An. funestus* and resistant *Culex quinquefasciatus.* Mosquito mortality, blood feeding inhibition and personal protection were compared between untreated nets, conventional alpha-cypermethrin treated nets (CTN) washed 20 times and LNs washed 0, 20 and 30 times.

**Results:**

In Phase I Interceptor LN demonstrated superior wash resistance and efficacy to the CTN. In the Phase II hut trial the LN killed 92% of female *An. gambiae* when unwashed and 76% when washed 20 times; the CTN washed 20 times killed 44%. The LN out-performed the CTN in personal protection and blood-feeding inhibition. The trend for *An. funestus* was similar to *An. gambiae* for all outcomes. Few pyrethroid-resistant *Cx. quinquefasciatus* were killed and yet the level of personal protection (75-90%) against *Culex* was similar to that of susceptible *An. gambiae* (76-80%) even after 20 washes. This protection is relevant because *Cx. quinquefasciatus* is a vector of lymphatic filariasis in East Africa. After 20 washes and 60 nights’ use the LN retained 27% of its initial insecticide dose.

**Conclusions:**

Interceptor LN meets the approval criteria set by WHO and is recommended for use in disease control against East African vectors of malaria and filariasis. Some constraints associated with the phase II evaluation criteria, in particular the washing procedure, are critically reviewed.

## Background

Long lasting insecticidal nets (LN) are an ideal method of preventing malaria in Africa south of the Sahara and many Asian countries [1,2]. They provide good protection against mosquito bites when used regularly [3], they are relatively cheap compared to other methods of personal protection [3], they are simple to distribute to communities [4], and they are popular [2]. The market for LN has burgeoned in recent years. To ensure that LN competing for the market are fit for purpose the World Health Organization, through its Pesticide Evaluation Scheme (WHOPES), has set standards of quality and efficacy that require threshold criteria to be met or surpassed, regardless of the type of netting material, mode of manufacture, pyrethroid compound and long lasting formulation. In order for any new LN to enter the market or be considered for tender by the main institutional buyers, it must attain WHOPES recommendation, which means in practice passing through a process of efficacy evaluation that has three phases [5,6]. During the Phase I evaluation the candidate LN must, after determination of regeneration time, show efficacy in mosquito bioassay over 20 standardized washes. During Phase II, standardized washed and unwashed LN are evaluated against host seeking, free flying mosquitoes in experimental huts. At this juncture the LN may attain interim recommendation from WHOPES [5]. Phase III evaluation requires the LN to meet performance criteria after 3 years of use with families living in malaria endemic areas [6].

Interceptor is a LN developed and produced by BASF Corporation. The netting is a polyester fibre coated with a proprietary polymer containing the insecticide alpha-cypermethrin at 200 mg/m^2^. The polymer binds to the fibre and can withstand multiple washings, the active ingredient diffusing in a controlled manner to the surface of the polymer coat to maintain insecticidal efficacy [7-9].

This paper reports upon Phase I laboratory and Phase II experimental hut evaluations of Interceptor LN. The laboratory bioassays were conducted in the U.K. using laboratory-reared pyrethroid-susceptible *Anopheles stephensi* mosquitoes. The hut trials were undertaken at the National Institute for Medical Research (NIMR) in Muheza, northeastern Tanzania, against wild free-flying *An. gambiae, An. funestus* and *Culex quinquefasciatus* mosquitoes.

## Methods

### Phase I

#### Nets and washing process

Interceptor LN nets were supplied by BASF Corporation (Ludwigshaven, Germany). The polyester net was treated with alphacypermethrin (coated onto filaments) at a target dose of 6.7 g AI/kg of netting material for 75-denier yarn, corresponding to 200 mg of alpha-cypermethrin per square metre of the polyester fabric (with a tolerance limit of ± 25%). Polyester nets of the same denier and source were treated by hand with an aqueous solution of alpha-cypermethrin (Fendona 10SC, BASF) at target dosages of 25 mg/m^2^ (hereafter CTN25) and 200 mg/m^2^ (CTN200) for use as positive controls. The washing procedure for Phase I testing of LNs followed the WHOPES guidelines [5,6]. Netting pieces (25 cm × 25 cm) were subjected to standardized washing for intervals of 5, 10, 15 and 20 times, with a one day interval between washes, using WHO-approved soap solution (Savon de Marseille) at 2g/L in deionized water at 30°C for 10 minutes in a shaker water bath set at 155 movements per minute. Pieces were then rinsed twice for 10 minutes in clean water.

#### Three minute exposure (ball) bioassays

Netting pieces were fixed to a metal frame consisting of two interlocking rings of 11.5 cm diameter [5,10]. Ten 2–5 day old unfed female *Anopheles stephensi* (Beech: pyrethroid-susceptible strain) mosquitoes were exposed for 3 minutes then transferred to holding cups and supplied with a 10% glucose solution. The number knocked down was recorded after 60 minutes and the number dead after 24 hours. Test conditions were 25 ± 2°C and 80 ± 5% RH throughout.

#### Median knockdown time bioassay

In a separate series of assays, eleven 2–5 day old unfed females were introduced into the netted frame, and as each mosquito was knocked down it was removed using an aspirator. Knockdown was defined as either collapsed against the netting or fallen to the base, and not moving. The time for the median mosquito (6th) to be knocked down was the end point of the test [5,10].

#### Chemical analysis

Alpha-cypermethrin content of unwashed and washed nets was determined from net samples measuring 5 cm × 5 cm using the method described by Yates *et al.*[11]. Alpha-cypermethrin was extracted using acetonitrile and injected onto HPLC (Dionex Summit, Camberly, Surrey, UK), separated on a 120Å column, eluted with a 9:1 solution of water:acetonitrile and passed through a PDA-100 detector at 275 nm. From the calibration curve the amount of alpha-cypermethrin on the netting pieces was estimated and the dosage per m^2^ calculated.

### Phase II

#### Study area and experimental huts

The experimental hut trial was conducted at the NIMR Field Station at Zeneti, Muheza, in northeastern Tanzania (5°13'S and 38°39'E) where *An. gambiae s.s.* and *An. funestus* are the major malaria vectors, and *Culex quinquefasciatus* is also common. *An. gambiae s.s.* at the Zeneti site are susceptible to pyrethroids but *Cx. quinquefasciatus* showed resistance, conferred by enhanced oxidase and site insensitivity mechanisms [12]. Confirmation of the resistance status of the wild adult mosquitoes was established during the trial by testing with alpha-cypermethrin 0.05% papers in WHO test kits [13].

The huts were constructed to a design described by WHO [13], based on the original verandah-hut design developed in Tanzania [14,15]. Minor modifications included a reduced eave gap of 2 cm, a ceiling, and a concrete floor surrounded by a water filled moat. The working principle of these huts has been described previously [10,16]. In brief, the huts had open eaves with veranda traps and window traps on each side. Two of the verandas were closed to capture any mosquitoes that exited via the eaves, and the two other verandas were left open so mosquitoes could enter the huts through the eaves. Each night’s collection inside the two screened veranda traps was multiplied by two and added to the room and window trap collections; the multiplication was designed to adjust for unrecorded escapes through the two open sides. At the end of each week the north and south verandas were closed and east and west verandas opened, or vice versa, to compensate for possible selective exiting in one compass direction.

#### Net preparation

The Interceptor LN, untreated nets and alpha-cypermethrin insecticide (Fendona 10SC) were supplied by BASF. All nets were 75 denier polyester and measured 2 m (L)×1.2 m (W) × 1.5 m (H). To simulate wear and tear a total of six 4 cm × 4 cm holes were cut into each net (two holes on each side and one hole at each end). The target concentration of alphacypermethin on the LNs and CTNs was 200 mg/m^2^. The LNs and CTNs were washed according to WHO Phase II washing protocols [5]. Each net was washed individually in 10 litres of tap water containing 2 g/litre of soap ('Savon de Marseille’), subjected to 20 rotations per minute for 6min during a 10min immersion, then rinsed twice. The interval between washes was 1 day which is the established regeneration time for Interceptor LN [9]. The washing schedule was stepped to ensure that the final wash of all treatment arms was completed on the same day.

The CTN washed to the 'point of insecticide exhaustion’ served as a positive control against which to assess Interceptor LN performance. The point of insecticide exhaustion, as defined by WHO, is the point at which the net causes less than 80% mortality and 95% knock down in WHO cone bioassays conducted after each wash [5]. Determination of the 'point of exhaustion’ was carried out by exposing unfed *An. gambiae s.s.* Kisumu in 10 replicates of 5 mosquitoes after each wash interval on the five panels of each net. Exposure was for 3 min, knockdown was scored after 60 min and mortality was scored 24 h later. The mortality on the alpha-cypermethrin CTN fell below 80% after 20 washes. On this occasion we opted to use a CTN washed 20 times as an appropriate control for the LN washed 20 times since the two treatment arms were both washed the same number of times.

#### Experimental hut study design

The following five treatment arms were tested in the huts:

1. Unwashed Interceptor LN

2. Interceptor LN washed 20 times

3. Interceptor LN washed 30 times, in accordance with the manufacturer’s claim for wash fastness

4. Polyester net conventionally treated with alpha-cypermethrin at 200 mg/m^2^ and washed 20 times

5. Untreated unwashed polyester net

The primary outcomes were:

1. Deterrence – the reduction in entry into treatment hut relative to the control huts (i.e. those containing untreated nets);

2. Treatment-induced exiting - the proportion of mosquitoes found in exit traps of treatment huts relative to the same proportion in control huts;

3. Mortality - the proportion of mosquitoes killed relative to the total catch size;

4. Overall killing effect - the numbers killed by a treatment relative to the untreated control, as derived from the formula

$$\mathrm{killing}\;\mathrm{effect}\;\left(\%\right)=100\frac{\left(\mathrm{Kt}-\mathrm{Ku}\right)}{\mathrm{Tu}}$$

where *Kt* is the number killed in the huts with treated nets, *Ku* is the number dead in the huts with untreated nets, and *Tu* is the total entering the huts with untreated nets.

1. Blood-feeding inhibition - the proportional reduction in blood feeding in huts with treated nets relative to controls with untreated nets

2. Personal protection - the reduction in mosquito biting by treated nets relative to untreated nets, as derived from the formula

$$\%\;\mathrm{personal}\;\mathrm{protection}\;=100\frac{\left(\mathrm{Bu}-\mathrm{Bt}\right)}{\mathrm{Bu}}$$

where *Bu* is the total number blood-fed mosquitoes in the huts with untreated nets, and *Bt* is the total number blood-fed in the huts with treated nets.

Each morning dead and live mosquitoes were collected from the verandahs, room and window traps. Live mosquitoes were provided with 10% sugar solution. Delayed mortality was recorded after 24h. Mosquitoes were identified to species and gonotrophic status was recorded as unfed, blood-fed, semi-gravid or gravid. Random samples of *An. gambiae s.l.* (n = 60) were identified to species by PCR [17].

The criteria for efficacy was that the Interceptor LN washed 20 times should perform equal to or better than the CTN washed until just before exhaustion. Twenty washes is set by WHO as an approximate number of washes a LN is likely to incur during its lifetime.

The trial took place between May and August 2006. The treatment arms were rotated twice through each hut according to a Latin Square design. A treatment was assigned at random to a particular hut for 6 nights’ observation before being transferred to the next hut. Between 19:30 and 6:30 hours adult male volunteers slept on beds under the nets. The same five sleepers were rotated through the huts on consecutive nights. Six nets were available per treatment arm and each net was tested on consecutive nights during the six-night rotation. At the end of the weekly rotation the huts were cleaned and aired for one day before starting the next rotation. Data were collected for 60 nights.

#### Chemical analysis

Netting samples were taken for determination of alpha-cypermethrin content by HPLC on three occasions: before washing, after completion of the washes, and after conclusion of the trial as described by WHO [5]. Four netting pieces, each measuring 10 cm × 10 cm, were cut from the sides, end and top before and after washing from a 7th net taken from each study arm (these nets were not used in the hut trial), while a net taken from the huts from each study arm was sampled at the end of the trial. HPLC analyses were carried out on each piece as described for Phase I assays, the average amount of alpha-cypermethrin estimated and the dosage per m^2^ calculated.

### Ethical clearance

Ethical clearance was obtained from the Ethics Committees of the National Institute for Medical Research Tanzania (Ref: NIMR/HQ/R.8a/Vol X/86), and the London School of Hygiene and Tropical Medicine (Ref: 8589). Written informed consent was obtained from all volunteers participating in the study. The risks of malaria were explained and all volunteers were provided with chemoprophylaxis. During the trial each volunteer was monitored daily for fever or possible adverse effects due to the LNs or CTNs.

### Analysis

The principal aim was to compare the efficacy of Interceptor LN washed 0 and 20 times to a CTN washed until 'exhaustion’. The key outcomes were the overall proportions of mosquitoes blood-feeding or dying relative to the untreated control. Logistic regression was used to estimate proportional outcomes of treatments (mortality, blood-feeding, exiting), and negative binomial regression was used to analyze counts of mosquitoes entering the huts (personal protection, overall insecticidal effects), after adjusting for clustering by day and for variation between individual sleepers and hut position. Laboratory bioassay data was analysed using logistic regression. Median knock down tests and chemical analysis was analysed using analysis of variance.

## Results

### Phase I - laboratory tests

**Figure 1:**
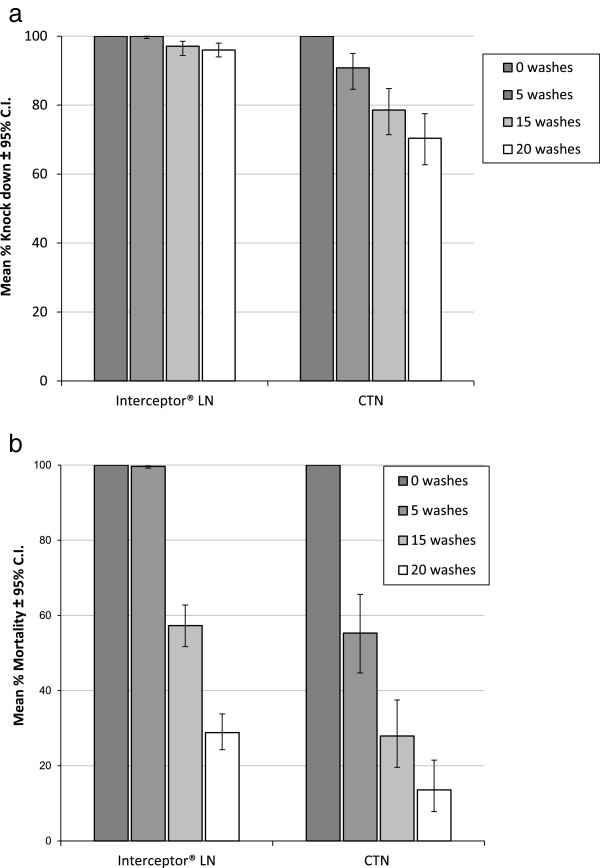
**Response of *Anopheles stephensi *** **exposed in 3 minute ball bioassays to Interceptor LN and alpha-cypermethrin CTN25: a. Knockdown. b. Mortality.**

*Ball bioassay tests*: Bioassay tests were done on Interceptor LN and CTN25 (25 mg/m^2^). The percentage knockdown of *An. stephensi* decreased from 100% to 70% on exposure to the CTN25 washed 0 and 20 times (p = 0.01) and from 100% to 96% on exposure to the LN washed 0 and 20 times (p = 0.03) (Figure 1a). Percentage mortality was 55% on the CTN25 and 99% on the LN after 5 washes, and decreased to 14% and 29% respectively after 20 washes (p = 0.01) (Figure 1b).

**Figure 2:**
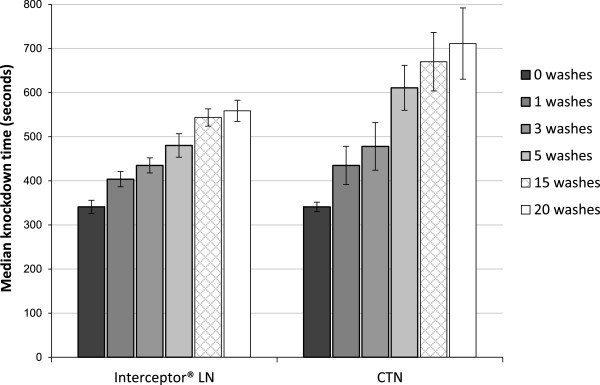
**Median time to knockdown of *Anopheles stephensi *** **exposed in 3 minute ball bioassays to Interceptor LN and alpha-cypermethrin CTN25.**

*Median knock down tests (MKDT)*: After each sequence of washes median knockdown time was extended, indicating the removal of surface insecticide. At each wash point MKDT took longer on the CTN25 than on the Interceptor LN indicating a lower surface concentration of insecticide on the CTN25 (p = 0.01) (Figure 2). There was correlation between percentage knockdown and the dosage of insecticide remaining on the netting after washing (r^2^= 0.46, p < 0.001) and between percentage mortality and dosage remaining (r^2^= 0.61, p = 0.001).

**Figure 3:**
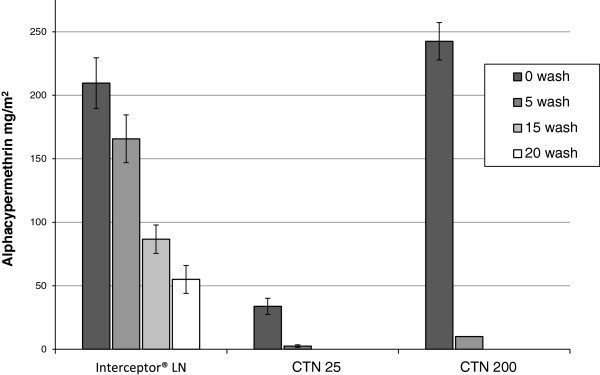
Chemical analysis of Phase I nets.

*Chemical analysis*: The alpha-cypermethrin content of the Interceptor LN decreased by 21% after 5 washes, from 209 mg/m^2^ to 166 mg/m^2^, and by 74% after 20 washes to 55 mg/m^2^ (Figure 3). The content of the CTN25 and CTN200, initially treated with 25 mg/m^2^ and 200 mg/m^2^, fell by 93% and 96% respectively after just 5 washes and by the 15th wash no alpha-cypermethrin was detectable by HPLC on either net.

### Phase II - experimental hut trial

#### Insecticide susceptibility

Susceptibilty tests using WHO kits confirmed full susceptibility to alphacypermethrin in *An gambiae* (100% mortality, N=351) and *An funestus* (100% mortality, N=401). *Culex quinquefasciatus* were resistant to pyrethroid (52% mortality, N=234) and the ratio of time to 50% knockdown compared to a laboratory susceptible strain (TPRI) was 5.1 (95% confidence interval: 4.2 - 6.0).

#### Mosquito entry into the huts

A total of 1,836 female mosquitoes were collected over the 66 nights of the trial (Table 1). These consisted of 834 (45.4%) *Anopheles gambiae*, 440 (24.0%) *An. funestus* and 562 (30.6%) *Culex quinquefasciatus.* The mean number caught per night was 12.6 *An. gambiae*, 6.7 *An. funestus* and 8.5 *Cx. quinquefasciatus*. There was no clear evidence of deterred entry associated with any of the treatments (Table 1). Relative to the untreated net there were fewer *An. gambiae* in huts with the unwashed LN and LN washed 20 times but this trend was not apparent for the other species or for the CTN treatment.

**Table 1:** Number of wild mosquitoes entering the experimental huts during the trial of Interceptor LN

	**Untreated net 0 W**	**Interceptor 0 W**	**Interceptor 20 W**	**Interceptor 30 W**	**CTN 20 W**
***Anopheles gambiae***					
Total females caught	171	134	122	183	224
Average catch per night	2.8^a^	2.2^b^	2.0^b^	3.0^a^	3.7^c^
***Anopheles funestus***					
Total females caught	81	68	87	79	125
Average catch per night	1.3^a^	1.1^a^	1.4^a^	1.3^a^	2.1^b^
***Culex quinquefasciatus***					
Total females caught	95	95	106	106	160
Average catch per night	1.6^a^	1.6^a^	1.8^a^	1.8^a^	2.7^b^

Numbers in the same row sharing a letter superscript do not differ significantly (P > 0.05).

#### Blood feeding inhibition and personal protection

**Figure 4:**
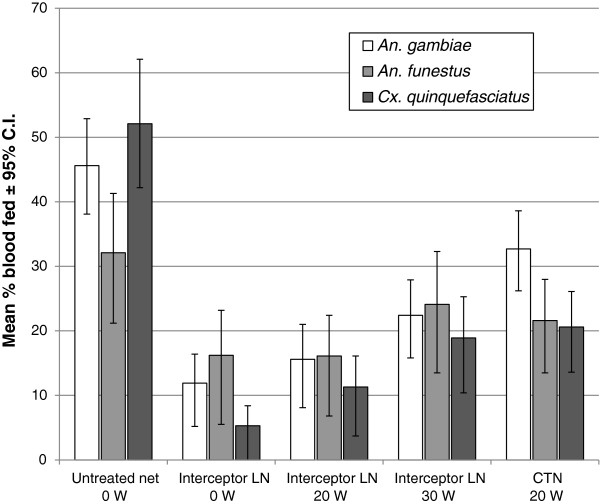
Blood feeding success in experimental huts with Interceptor LN and alpha-cypermethrin CTN versus untreated control.

Blood feeding rates in huts with the holed untreated nets ranged from 32.1% for *An. funestus*, 45.6% for *An. gambiae* and 52.1% for *Cx. quinquefasciatus* (Figure 4). Relative to the untreated nets the blood feeding rates through the unwashed LN was 2.0 (32.1%/16.2%) times less for *An. funestus*, 2.9 (45.6%/11.9%) times less for *An. gambiae* and 9.8 (52.1%/5.3%) times less for *Cx. quinquefasciatus*. The percentage blood feeding inhibition associated with unwashed LN was 49.6%, 73.8% and 89.8% respectively against these species (Table 2). The blood feeding rates with the LN washed 20 times did not differ significantly from the unwashed LN, and the levels of blood feeding inhibition also hardly changed. After 30 washes there were significant increases in percentage blood feeding relative to the unwashed LN; however, even after 30 washes the blood-feeding rates were still 50% less for *An. gambiae*, 64% less for *Cx. quinquefasciatus* and 25% less for *An. funestus* for the LN relative to the untreated net (Table 2). The percentage blood feeding inhibition associated with the CTN washed 20 times was significantly less than for the LN washed 20 times with respect to *An. gambiae* (p=0.01) and *Cx. quinquefasciatus* (p=0.01) but not to *An. Funestus,* owing to the lower abundance.

**Table 2:** Blood-feeding inhibition and personal protection rates due to Interceptor LN and CTN in the experimental hut trial

	**Untreated net 0 W**	**Interceptor 0 W**	**Interceptor 20 W**	**Interceptor 30 W**	**CTN 20 W**
***Anopheles gambiae***					
Total blood fed	78	16	19	41	73
% Blood feeding inhibition	0^a^	73.8^b^	65.8^bc^	50.9^c^	28.3^d^
% Personal Protection	0^a^	79.5^b^	75.6^b^	47.4^c^	6.4^a^
***Anopheles funestus***					
Total blood fed	26	11	14	19	27
% Blood feeding inhibition	0^a^	49.6^b^	49.8^b^	24.9^ab^	32.7^ab^
% Personal Protection	0^a^	57.7^b^	46.2^b^	26.9^b^	0^a^
***Culex quinquefasciatus***					
Total blood fed	49	5	12	20	33
% Blood feeding inhibition	0^a^	89.8^b^	78.3^bc^	63.7^cd^	60.4^d^
% Personal Protection	0^a^	89.8^b^	75.5^bc^	59.2^c^	32.7^d^

Numbers in the same row sharing a letter superscript do not differ significantly (P > 0.05).

Personal protection examines the relative number of mosquitoes that blood feed in the presence of treated nets compared to untreated nets. Because there was little or no deterrence associated with the alpha-cypermethrin treated nets the levels of percentage personal protection were quite similar to percentage blood feeding inhibition of each treatment. Personal protection with the unwashed LN and the LN washed 20 times was over 75% against *An. gambiae* and *Cx. quinquefasciatus*, and over 45% against *An. funestus*. There was no evidence of personal protection from the CTN washed 20 times against the anophelines.

#### Mortality and overall killing effect

**Figure 5:**
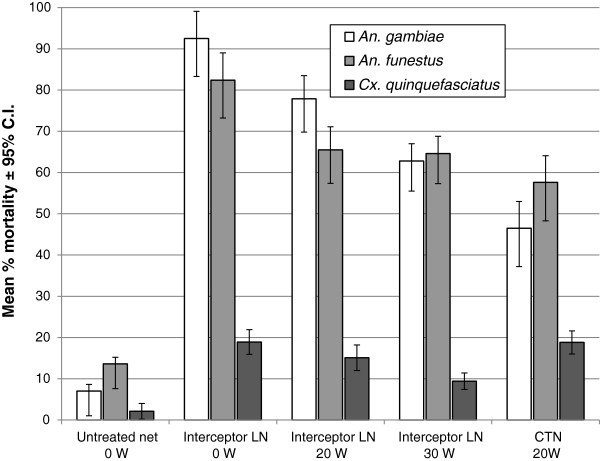
Mortality rates in experimental huts with Interceptor LN and CTN versus untreated control.

The unwashed Interceptor LN killed 91.9% of *An. gambiae* that entered the hut (Figure 5, Table 3). This fell to 76.2% mortality after 20 washes and to 60.0% after 30 washes. This observed decline in efficacy was statistically significant (p=0.001). The mortality induced by Interceptor LN washed 20 times was significantly higher than the mortality induced by the alpha-cypermethrin CTN washed 20 times (p=0.01). *An. funestus* showed similar trends but with lower rates of mortality compared to *An. gambiae* for the Interceptor treatments (Figure 5, Table 3). The difference in mortality between the Interceptor LN washed 20 times and the CTN washed 20 times was not significant. The LNs and ITNs killed only 8-20% of *Cx. quinquefasciatus*, this species being resistant to pyrethroids.

**Table 3:** Mortality and overall killing effect of Interceptor LN in the experimental hut trial

	**Untreated net 0 W**	**Interceptor 0 W**	**Interceptor 20 W**	**Interceptor 30 W**	**CTN 20 W**
***Anopheles gambiae***					
Total females dead	12	124	95	115	103
% corrected mortality	0^a^	91.9^b^	76.2^c^	60.0^d^	44.2^e^
% Overall killing effect	0^a^	70.4^b^	52.2^d^	64.8^bc^	57.2^cd^
***Anopheles funestus***					
Total females dead	11	56	57	51	72
% corrected mortality	0^a^	79.6^b^	60.1^c^	59.0^c^	50.9^c^
% Overall killing effect	0^a^	64.3^b^	65.7^b^	57.1^b^	87.1^c^
***Culex quinquefasciatus***					
Total females dead	2	18	16	10	30
% corrected mortality	0^a^	17.2^b^	13.3^b^	7.5^c^	17.0^b^
% Overall killing effect	0^a^	17.2^bc^	15.1^b^	8.6^b^	30.1^c^

Numbers in the same row sharing a letter superscript do not differ significantly (P > 0.05).

The overall killing effect of the Interceptor LN against the two Anopheline species ranged between 70-50% over 0 and 30 washes, but killed less than 20% of *Cx. quinquefasciatus* even when unwashed.

#### Exiting rates

The majority of *An. gambiae* and *An. funestus* (>86%) naturally exited the huts before dawn and were collected from the verandah and window traps (Table 4). The proportions exiting huts with LN were rather higher than from huts with untreated nets. In contrast to the Anophelines, around 50% of *Cx. quinquefasciatus* remained in the hut each morning (Table 4). However, in huts with pyrethroid nets, between 81% and 96% of *Culex* were induced to exit into the traps before dawn.

**Table 4:** Percentage of mosquitoes exiting huts into verandah and window traps in the Interceptor LN experimental hut trial

	**Untreated net 0 W**	**Interceptor 0 W**	**Interceptor 20 W**	**Interceptor 30 W**	**CTN 20 W**
***Anopheles gambiae***					
Total females exiting	147	116	113	174	207
% Exiting	86.0^a^	86.6^a^	92.6^ab^	95.6^b^	92.8^ab^
***Anopheles funestus***					
Total females exiting	70	65	86	76	120
% Exiting	86.4^a^	95.6^ab^	98.9^ab^	96.2^ab^	96.0^ab^
***Culex quinquefasciatus***					
Total females exiting	51	91	94	94	130
% Exiting	54.0^a^	95.8^b^	88.7^bc^	88.7^bc^	81.2^cd^

Numbers in the same row sharing a letter superscript do not differ significantly (P > 0.05).

#### Chemical analysis and bioassays on nets

The alpha-cypermethrin content on the nets before and after washing is summarised in Table 5. Chemical analysis showed that the initial dosages of the active ingredients were below the expected dosages, both for CTN (147 mg/m^2^ instead of 200 mg/m^2^) and for LN (132-142 mg/m^2^ instead of 200 mg/m^2^). The tolerance limit of alpha-cypermethrin on the LN is 200 mg AI per m^2^ ± 25% [6] and, as such, the alpha-cypermethrin content in unwashed Interceptor was found to be close to the lower tolerance limit. After 20 and 30 washes the concentrations on the LN had decreased to 41 mg/m^2^ and 21 mg/m^2^ respectively. In contrast, the concentration in alpha-cypermethrin CTN was only 1-3 mg/m^2^ after 20 washes. After 60 days of use in experimental huts, further decrease of alpha-cypermethrin was not evident.

**Table 5:** Chemical analysis of alpha-cypermethrin on the Interceptor LN and CTN before and after washing and at the end of the experimental hut trial

**Treatment**	**Concentration of alpha-cypermethrin (mg/m**^**2 **^**± std dev)**		
	**Before washing**	**After washing**	**After hut trial**
Untreated net	-	-	0
Interceptor LN unwashed	147 ± 2	-	140 ± 5
Interceptor washed 20 times	132 ± 4	41 ± 5	36 ± 3
Interceptor washed 30 times	-	21 ± 1	21 ± 1
CTN washed 20 times	144 ± 17	1.2 ± 0.4	1.0 ± 0.6

Cone bioassay tests were carried out on these same net samples before and after washing. Before washing the LN and CTN treatments both recorded 100% mortality and 100% knockdown in cone tests. After 20 washes the LN recorded 100% mortality and 100% knockdown and the CTN recorded 68% mortality and 33% knockdown.

## Discussion

The WHO Phase II experimental hut trial demonstrated that the blood-feeding inhibition (the proportional reduction in biting / blood feeding) and percentage mosquito mortality induced by Interceptor LN washed 20 times was superior to that of the CTN washed to exhaustion, and therefore the LN fulfilled the WHOPES criterion of a long lasting insecticidal net [5]. On the basis of these results, which formed part of an official WHOPES evaluation, Interceptor LN received interim recommendation as an approved LN [9]. Earlier in 2013 the WHOPES guidelines for testing of LN were revised to include as a positive control a WHOPES-recommended LN with similar specifications to the candidate LN in terms of insecticide, treatment technique, netting material, and washing frequency (0 and 20 times) [6]. The revised guidelines were issued after the current trial and, indeed, Interceptor now constitutes a LN appropriate to use as a positive control against new candidate LN. Because more brands of LN are being submitted to WHO for recommendation, one of the purposes of the revision is to demonstrate that new candidate LN match or exceed the standards set by previously approved LN such as Interceptor. Recent WHOPES trials have included both a reference LN washed 20 times and a CTN washed to exhaustion as comparison arms to ensure that equivalence or superiority of the reference LN to the CTN is being maintained.

The Phase I laboratory bio- and chemical assays confirmed that the Interceptor LN insecticide binding process imparts strong wash-retention characteristics. The laboratory washing regime stripped the alpha-cypermethrin from the conventionally treated net within a few washes (surface content falling from 200 to 10 mg/m^2^) and to levels undetectable by HPLC within 15 washes. However, in bioassays on the same CTN (washed 15 times) the median mosquito was knocked down after just 11 minutes exposure and mortality reached 28% in the 3 minute bioassays. A similar finding was observed in the Phase II experimental hut trials: the CTN washed 20 times had a surface concentration of only 1.2 mg/m^2^ and yet this net was still able to kill 44% of *An. gambiae* and 51% of *An. funestus* that entered and came into contact with it. The only explanation is that pyrethroids such as alpha-cypermethrin must have a strong affinity to the polyester netting fibres so that even after vigorous washing a thin layer of pyrethroid, virtually undetectable by HPLC yet sufficiently bioactive to induce knockdown and mortality, must still remain bound to the fibres.

The performance of the CTN and the level of mortality and knockdown it induced after washing, while being surprising, were still not comparable to those of the LN. Interceptor LN retained a surface concentration of over 40 mg/m^2^ after 20 washes (30 times greater than the CTN’s) and induced significantly higher levels of mortality than the CTN washed the same number of times. The comparison does, however, raise some issues and limitations concerning the WHOPES Phase II process. The Phase II preparation is designed to mimic the washing practices of net owning families; it cannot mimic the myriad ways in which insecticide is removed from the nets during a lifetime of use. An important source of removal during Phase III must be the abrasion a net is subjected to daily during 3 years of household use. In contrast, the only abrasion a net is subjected to during Phase II is the stirring and mashing during the 20 preparatory washes, and the 30–40 days of use during the hut trial. For this reason a WHOPES Phase II cannot anticipate or predict the outcome of evaluation after 3 years of household use. Only a WHOPES Phase III - in which nets are distributed to householders and re-gathered for testing after 3 years - can show whether a LN really does justify its WHO recommendation. For this reason the Phase II trial should only lead to an interim WHO recommendation. The WHO process is best seen as a series of gates with one phase setting a standard and leading to the next phase, rather than being predictive of the outcome of the next phase. The reality of this is demonstrated in the WHO report of the Phase III evaluation of Interceptor: it achieved the efficacy criteria of a true LN after 3 years of household use, whereas the CTN fell short of the efficacy criteria within just 1 year of use [9]. The failure of the CTN within a year of Phase III would not be predicted by the relative mortality shown by the CTN washed to exhaustion and Interceptor LN during Phase II. This raises the question of whether the Phase II preparatory procedure should include an accelerated abrasion process between washes that better mimics the wear and tear that a net is subjected to during a lifetime of use.

*Culex quinquefasciastus* and *Anopheles gambiae* were fully capable of feeding through holed untreated nets but when the nets were treated with pyrethroid the proportion that fed was reduced substantially, from 50% to 10-20%. The level of personal protection from the LN was 75-80% for *An. gambiae* and 75-90% for *Cx. quinquefasciatus*. The results for *An. gambiae* were expected, the results for *Cx. quinquefasciatus* results were not. This is because Tanzanian *Cx. quinquefasciatus* are highly resistant to pyrethroids due to site insensitivity and oxidase mechanisms [12], less than 20% are killed by the LN or CTN, and yet very few succeeded in blood feeding. In West Africa too, pyrethroid resistant *Cx quinquefasciatus* struggle to feed through holed LNs or ITNs [18,19]. In contrast, where *An. gambiae* has developed high level resistance due to a combination of *kdr* plus cytochrome P450 mechanisms [20] the proportion that manage to blood feed through holed LNs may increase to 60% or more [21,22]. LN seem to lose their capacity to protect when anophelines become highly resistant yet seem to retain capacity to protect when *Cx quinquefasciatus* becomes resistant. The reason for the difference between genera is not clear but may be due to behavioural differences around the net. This is particularly relevant to East Africa because *Cx. quinquefasciatus* is an important vector of lymphatic filariasis there [23]. The evidence from the present trial is that an LN will provide protection against *Cx. quinquefasciatus*-borne filariasis despite the species being resistant to pyrethroids.

## Conclusion

Consequent to this Phase II experimental hut trial Interceptor LN obtained interim approval from WHO and has since achieved full recommendation after Phase III trials. It is the first LN to contain the pyrethroid alpha-cypermethrin and the third LN to obtain full WHOPES approval.

## Competing interests

The authors declare that they have no competing interests.

## Authors’ contributions

RM contributed to the study design, supervised the project, processed the data, carried out the analysis, interpreted the data and drafted the manuscript. SMM managed the project and entomological teams, participated in the design of the study, interpreted the data and contributed to drafting the manuscript. PKT and VM carried out trial and processed the collections and data. CM contributed to the study design and supervision of research, interpreted the data and critically reviewed the manuscript. MJK interpreted the data and critically reviewed the manuscript. MR contributed to study design, data analysis and interpretation and revised the manuscript. All authors read and approved the final manuscript.
